# TG-CSR: A human-labeled dataset grounded in nine formal commonsense categories

**DOI:** 10.1016/j.dib.2023.109666

**Published:** 2023-10-11

**Authors:** Henrique Santos, Alice M. Mulvehill, Ke Shen, Mayank Kejriwal, Deborah L. McGuinness

**Affiliations:** a Rensselaer Polytechnic Institute 110 8th St., Troy, NY 12180, USA; b University of Southern California 4676 Admiralty Way, Suite 1001 Marina del Rey CA, 90292, USA

**Keywords:** Commonsense benchmark, Large language models, Question answering, Human agreement, Human annotation

## Abstract

Machine Common Sense Reasoning is the subfield of Artificial Intelligence that aims to enable machines to behave or make decisions similarly to humans in everyday and ordinary situations. To measure progress, benchmarks in the form of question-answering datasets have been developed and published in the community to evaluate machine commonsense models, including large language models. We describe the individual label data produced by six human annotators originally used in computing ground truth for the Theoretically-Grounded Commonsense Reasoning (TG-CSR) benchmark's composing datasets. According to a set of instructions, annotators were provided with spreadsheets containing the original TG-CSR prompts and asked to insert labels in specific spreadsheet cells during annotation sessions. TG-CSR data is organized in JSON files, individual raw label data in a spreadsheet file, and individual normalized label data in JSONL files. The release of individual labels can enable the analysis of the labeling process itself, including studies of noise and consistency across annotators.

Specifications Table

**Table:** 

Subject	Artificial Intelligence
Specific subject area	Machine Common Sense Reasoning is the subfield of Artificial Intelligence that aims to enable machines to behave (or make decisions) similarly to humans in everyday situations.
Type of data	Table
How the data were acquired	TG-CSR prompts were manually created by humans following a documented methodology that grounded each prompt into one category from a formal theory of commonsense thinking. Individual labels for each prompt were produced by six human annotators according to a set of annotation instructions. Annotators were provided with spreadsheets containing the original TG-CSR prompts and asked to insert their labels in specific spreadsheet cells.
Data format	TG-CSR benchmark data (JSON) Individual raw label data (XLSX) Individual normalized label data (JSONL)
Description of data collection	Individual label data were collected during annotation sessions with six human annotators. The annotators worked on their own machines and in their own physical environment of choice. No labels were initially discarded. Labels that could not be interpreted (such as missing labels or wrong labels) were removed from the final datasets.
Data source location	Rensselaer Polytechnic Institute Tetherless World Constellation 110 8th St. Troy, NY USA 12180 University of Southern California Information Sciences Institute 4676 Admiralty Way Marina Del Rey, CA USA 90292
Data accessibility	TG-CSR benchmark data, including individual raw and normalized label data: https://doi.org/10.5281/zenodo.8279823
Related research article	H. Santos, K. Shen, A.M. Mulvehill, M. Kejriwal, D.L. McGuinness. 2023. A Theoretically Grounded Question Answering Data Set for Evaluating Machine Common Sense. *Data Intelligence*, forthcoming.

## 1 Value of the Data

• TG-CSR, to the best of our knowledge, is the first commonsense reasoning benchmark that is grounded in a formal theory of commonsense thinking. Therefore, it allows us to claim that we can evaluate specific representational areas of human common sense.
• Because TG-CSR includes the individual labels produced by the human annotators, it can be used in support of richer analysis of the labeling process itself, including studies of noise and consistency across annotators.
• AI Developers can use TG-CSR to train or evaluate their models across several select foundational commonsense representational areas.

## 2 Objective

Developing machines with commonsense reasoning (CSR) abilities is a longstanding challenge in the Artificial Intelligence (AI) community. To measure progress, benchmarks in the form of question-answering datasets have been proposed and published in the community to evaluate machine commonsense models, including large language models. Because there is no single definition of what human commonsense is, it is not trivial to effectively characterize a benchmark as being a CSR benchmark. However, one characteristic of common sense is high agreement among several humans. In previous work, we evaluated the usefulness of using nine reasoning representation categories (such as time, space, etc.) identified by Gordon & Hobbs [1] as fundamental to commonsense reasoning to construct datasets for use in measuring human annotator agreement on commonsense sentences [2]. In addition, we proposed several evaluation modalities and tasks for use in the development of commonsense benchmarks [3]. In this article, we describe the contents of the produced datasets that compose the Theoretically-Grounded Commonsense Reasoning (TG-CSR) [4] benchmark. Furthermore, we describe and release the individual human labels produced during the annotation sessions of TG-CSR.

## 3 Data Description

TG-CSR data is split into three major categories: benchmark data (prompts and compute ground truth), individual raw label data, and individual normalized label data. The benchmark data is the main TG-CSR dataset, which is composed of four contexts (``Vacationing Abroad'', ``Camping Vacation'', ``Bad Weather'', and ``Dental Cleaning''), each of which is presented in two task formats: multiple-choice (MC) and true/false (TF). The MC format is organized in five files, while the TF format is in two files. Each file has a purpose, as detailed in Table 1.

**Table 1:** Description of the TG-CSR benchmark data files and label data.

TG-CSR	
File	Description
[context]-mc-questions.json	All MC questions for a context. Each question has a unique assigned question ID within a context. File structure: {“context”: string, context description,“theme”: string, theme description, “questions”: array of: {“questionID”: integer, id of the question,“question”: string, question text}}
[context]-mc-questions-annotations.json	The assignment of the corresponding commonsense category to each question in a context, using the question ID. File structure: array of: {“questionID”: integer, id of the question,“category”: string, commonsense category}
[context]-mc-answers.json	All MC answers for a context. Each answer has a unique assigned answer ID within a context. File structure: array of: {“answerID”: integer, id of the answer, “answer”: string, answer text}
[context]-mc-answers-annotations.json	The assignment of the corresponding commonsense category to each answer in a context, using the answer ID. File structure: array of: { “answerID”: integer, id of the answer, “category”: string, commonsense category}
[context]-mc-ground_truth.json	The assignment of the computed ground truth for each question/answer pair within a context, using the question and answer IDs. File structure: array of: {“questionID”: integer, id of the question, “answerID”: integer, id of the answer,“gt”: integer, computed ground truth label}
[context]-tf-sentences.json	All TF sentences for a context. Each sentence refers to the original question/answer they were derived from using the question and answer IDs. File structure: {“context”: string, context description,“theme”: string, theme description,“sentences”: array of: {“questionID”: integer, id of the question,“answerID”: integer, id of the answer,“sentence”: string, sentence text}}
[context]-tf-ground_truth.json	The assignment of the computed ground truth for each sentence within a context, using the question and answer IDs. File structure: array of: {“questionID”: integer, id of the question,“answerID”: integer, id of the answer,“gt”: string, computed ground truth label}
**TG-CSR Annotations**	
**File**	**Description**
tg-csr_raw_labels.xlsx	Spreadsheet with individual raw label data produced by six annotators.
[context]-mc.jsonl	Individual normalized label data for a context in the MC format. File structure: {“answerID”: string, id of the answer prepended with the initials of the context,“questionID”: string, id of the question prepended with the initials of the context,“category”: string, commonsense category,“labels”: array of: {“annotatorID”: string, id of the annotator,“label”: integer, normalized label}}
[context]-tf.jsonl	Individual normalized label data for a context in the TF format. File structure: {“answerID”: string, id of the answer prepended with the initials of the context,“questionID”: string, id of the question prepended with the initials of the context,“category”: string, commonsense category,“labels”: array of: {“annotatorID”: string, id of the annotator, “label”: integer, normalized label}}

The individual raw label data is organized in a spreadsheet file (tg-csr-raw_labels.xlsx) with a total of eight tabs, one for each context and task format. In each tab, the labels produced by human annotators are in columns titled A, B, C, D, E, and F. The labels are associated with the appropriate MC question/answer pair or TF sentence using the respective question and answer IDs. For convenience, the associated commonsense category is replicated in the column titled category.

The individual normalized label data is organized in jsonlines files (JSONL), one for each context and task format ([*context*]-mc.jsonl and [context]-tf.jsonl). This normalized data is directly derived from the raw data (and therefore has the columns of the raw data spreadsheets as JSON keys), where the MC 1–4 labels were converted to 0–1 labels. The TF labels remained unchanged. The details are presented in the following Section.

## 4 Experimental Design, Materials and Methods

For the TG-CSR benchmark [4] data, the MC format was developed first. Question/answer pairs about nine categories (Time, Goals, Scheduling, World States, Activities, Emotions, Physical Entities, Space, and Values) considered by Gordon & Hobbs to be fundamental to commonsense reasoning [1] were developed for four contexts (Vacationing Abroad, Camping Vacation, Bad Weather, and Dental Cleaning). From each question/answer pair in the MC format, a sentence was created by slightly revising the question text with the respective answer. Table 2 displays examples of questions and answers in the MC format and the converted sentence in the TF format, across all four contexts, in the "Time" category. The created prompts in both task formats were organized in the datasets listed in Table 1 (with the ground truth being produced next).

**Table 2:** Examples of ``Time'' questions in MC format and the respective conversion to a TF sentence for each context.

Context	MC Question	MC Answer	TF Sentence
Vacationing Abroad	What is a reasonable time to spend outside a hotel during a vacation?	Between 1 and 2 hours	While on vacation, between 1 and 2 hours is a reasonable time to spend outside of a hotel.
		Around 2/3 of the day	While on vacation, around 2/3 of the day is a reasonable time to spend outside of a hotel.
Camping Vacation	How long might it take to set up a small tent?	1 hour	It takes 1 hour to set up a small tent.
		12 hours	It takes 12 hours to set up a small tent.
Bad Weather	How many days in advance is a hurricane warning typically issued by the weather service?	10 days	The weather service typically issues a hurricane warning in advance by 10 days.
		1 day	The weather service typically issues a hurricane warning in advance by 1 day.
Dental Cleaning	Mary had her first dental cleaning in January. When should she book the next two dental cleaning appointments?	March	Mary had her first dental cleaning in January so she should book one of the next two dental cleaning appointments in March.
		December	Mary had her first dental cleaning in January so she should book one of the next two dental cleaning appointments in December.

### 4.1 Data Annotation

For producing the individual label data, a spreadsheet containing ten tabs was created for each context and task format. The first tab specified the context (e.g. Vacationing Abroad) and provided a Theme (a short background story possibly with specified goals or constraints) for the context. The additional nine spreadsheet tabs contained the prompts related to each of the nine selected commonsense categories. For the MC format, each category tab contained 2–5 questions, each with 8–12 answer options that were to be used for answering all questions in a tab. For the TF format, the converted TF sentences replaced the respective question/answer pair. A total of eight spreadsheets were created.

Six annotators, all affiliated with the organizations involved with the research, were provided with the above eight spreadsheets, one at a time. The annotators were also provided with written instructions. In these instructions, the annotators were asked to answer all questions in each tab in one session, to provide the start time and the end time of their session in the Theme and Context tab, and to read the content of the Theme and Context tab before answering the questions. Note that the tab names were anonymized so that the annotators were not biased by the category names. In the MC spreadsheets, the annotators were asked to rate how good a particular answer option fit the question using the following scale: 4 (very good fit), 3 (good fit), 2 (not sure), 1 (bad fit), and to provide feedback comments as needed. For the TF spreadsheets, the annotators were asked to answer T (True) or F (False) for all sentences and to provide feedback comments as needed.

### 4.2 Individual Label Data and Ground Truth

The labels from annotation sessions were used to create ground truth for the TG-CSR benchmark data (and organized in the ground truth files in Table 1). To compute the ground truth for each prompt, we essentially used the statistical mode (the value that appears most often across all labels of a prompt). For the TF task format, this calculation was trivial. For the MC format, because the labels were on a 1–4 scale, we first normalized the labels as such:

• Labels 1 (bad fit) and 2 (not sure) were converted to 0, indicating the answer is wrong for the question;
• Labels 3 (good fit) and 4 (very good fit) were converted to 1, indicating the answer is correct for the question.

After this step, we applied the statistical mode to compute the ground truth. The individual raw label data from the annotators (before normalization) was organized in the tg-csr-annotations.xlsx file. The individual normalized label data, in its turn, was organized in the JSONL files.

## Ethics Statements

All human annotators involved in this work have consented to the release of the produced individual label data. In addition, the annotators have been anonymized in the released datasets.

## CRediT authorship contribution statement

**Henrique Santos:** Conceptualization, Methodology, Software, Writing – original draft. **Alice M. Mulvehill:** Conceptualization, Methodology, Writing – original draft. **Ke Shen:** Validation, Writing – review & editing. **Mayank Kejriwal:** Conceptualization, Methodology, Writing – review & editing, Supervision. **Deborah L. McGuinness:** Supervision, Project administration.

## Data Availability

TG-CSR benchmark (Original data) (Zenodo)
